# A Z-Axis Quartz Cross-Fork Micromachined Gyroscope Based on Shear Stress Detection

**DOI:** 10.3390/s100301573

**Published:** 2010-03-01

**Authors:** Liqiang Xie, Xuezhong Wu, Shengyi Li, Haoxu Wang, Jianbin Su, Peitao Dong

**Affiliations:** College of Mechatronics Engineering and Automation, National University of Defense Technology, Changsha, 410073, Hunan Province, China; E-Mails: xzwu@nudt.edu.cn (X.W.); syli@nudt.edu.cn (S.L.); whx152@gmail.com (H.W.); sujianbin1983@163.com (J.S.); dongpeitao@nudt.edu.cn (P.D.)

**Keywords:** quartz micromachined gyroscope, cross-fork, shear stress detection, inertial sensor, Coriolis’ force, anisotropic etching, aperture mask

## Abstract

Here we propose a novel quartz micromachined gyroscope. The sensor has a simple cross-fork structure in the x-y plane of quartz crystal. Shear stress rather than normal stress is utilized to sense Coriolis’ force generated by the input angular rate signal. Compared to traditional quartz gyroscopes, which have two separate sense electrodes on each sidewall, there is only one electrode on each sidewall of the sense beam. As a result, the fabrication of the electrodes is simplified and the structure can be easily miniaturized. In order to increase sensitivity, a pair of proof masses is attached to the ends of the drive beam, and the sense beam has a tapered design. The structure is etched from a z-cut quartz wafer and the electrodes are realized by direct evaporation using the aperture mask method. The drive mode frequency of the prototype is 13.38 kHz, and the quality factor is approximately 1,000 in air. Therefore, the gyroscope can work properly without a vacuum package. The measurement ability of the shear stress detection design scheme is validated by the Coriolis’ force test. The performance of the sensor is characterized on a precision rate table using a specially designed readout circuit. The experimentally obtained scale factor is 1.45 mV/°/s and the nonlinearity is 3.6% in range of ±200 °/s.

## 1. Introduction

Over the past decades, micromachined gyroscopes for measuring rate or angle of rotation have be used in various inertia measurement fields, due to their small size, low cost, batch fabrication, and high reliability. They can companion with micromachined accelerometers to provide attitude information for inertial navigation or stability control [1]. For the improvement of performance, a number of researches have emphatically focused on the enhancement in material, fabrication and structural design [2–5]. At present, micromachined gyroscopes can be roughly divided into two kinds: silicon gyroscopes and quartz gyroscopes. The development of semiconductor technology has led to a rapid improvement in silicon gyroscopes. Moreover, for convenient excitation and detection, the piezoelectric effect promotes the prosperity of quartz gyroscopes.

Generally, a quartz gyroscope consists of a tuning fork structure along the *y* crystal axis of quartz. The tuning fork vibrates in *x* direction as drive mode and in *z* direction as sense mode [6–13]. Thus the sense mode generates charges on *x* surfaces (sidewalls) of the tuning forks by the normal stress along the *y* axis induced by Coriolis’ force in *z* direction. According to the quartz’s piezoelectric equation, the polarities of charges on one sidewall are different for the opposite direction of stress between front region and rear region [14]. Thus two parallel independent sense electrodes on each sidewall are required to gather charges [13,14]. However, the complicated electrode patterns on sidewalls are hard to fabricate by the two-dimensional micromachining technology, and imperfect fabrication seriously affects the performances of the sensors. Therefore, many researchers are eager to develop approaches to reduce the difficulty of the fabrication process and to improve the sensors’ performance. For example, the Nihon Dempa Kogyo Company in Japan simplifies the sense electrodes on sidewalls by using two monolithic quartz wafers bonded with reverse electrical axes to each other [15,16]. Kenji Sato divides the structure into drive part and sense part to change the vibratory direction of sense mode. In this way, the sense electrodes become easier to fabricate [17].

Our work focuses on the design of a novel quartz gyroscope structure to avoid complicated sense electrode patterns. The structure we propose here consists of a cross-fork formed by a drive beam and a sense beam. The drive mode of the gyroscope is designed as vibrating in the *x* direction. However, the sense mode is designed as vibrating in the *y* direction, and the *x*-*y* shear stress in the beam induced by Coriolis’ force in *y* direction can be detected by a pair of sense electrodes on sidewalls. Therefore, shear stress detection can simplify the structure and simplify the production of the sidewall electrodes. The design, fabrication, and characterization of the z-axis quartz cross-fork micromachined gyroscope are discussed in the paper.

## 2. Design Concept

**Figure 1.**
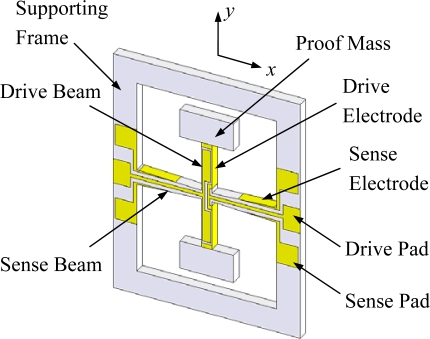
The gyroscope’s structure.

The schematic sketch of the presented z-axis quartz cross-fork gyroscope is shown in Figure 1. The sensor has a simple structure in the *x*-*y* plane of the crystal, consisting of a drive beam, a sense beam and two proof masses. The drive beam and the sense beam form a cross-fork structure that connects the supporting frame at the ends of the sense beam. The electrodes are laid on the surface of the beams and the pads are arranged on the surface of the supporting frame, which is fixed to a pedestal. The overall structure is symmetrical relative to the *x* and *y* axes.

### 2.1. Working Principle

**Figure 2.**
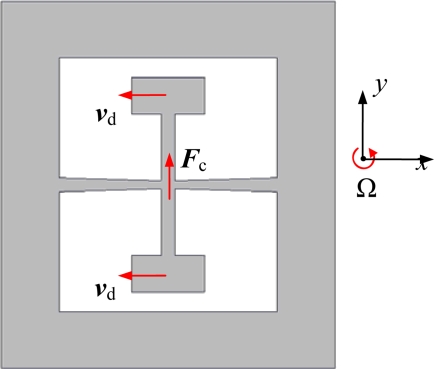
Schematic diagram of the working principle.

The working principle of the gyroscope is based on Coriolis’ force effect. A schematic diagram of the working principle is shown in Figure 2. All the beams vibrate in the structure plane perpendicular to the rate axis. The drive mode is the in-phase vibration of the two proof masses in *x* direction and the vibratory velocity is *v*_d_. When the whole structure is rotating about the *z* axis at a constant angular rate Ω, the sense beam is forced to vibrate in the *y* direction by Coriolis’ force *F*_c_ from the drive beam and this vibration is sense mode. The value of *F*_c_ is deduced as (1).

(1) $$F_{c}=-{2\mathrm{mv}}_{d}\times \Omega$$

where *m* is the equivalent mass of the proof masses.

According to the piezoelectric effect, charges are generated on the sidewalls of the sense beam by shear stress due to the vibration of the sense beam. Thus, we can measure changes of the charges to sense the angular rate Ω.

### 2.2. Electrodes Design

According to the drive mode and sense mode, the electrodes of the drive beam and sense beam are designed by the piezoelectric equations as in (2) [18].

(2) $$\left\{ \begin{array}{l} D=\varepsilon^{T}\cdot E+d\cdot T\\ S=d^{t}\cdot E+s^{E}\cdot T \end{array}\right.$$

***S***, ***T*** are elastic field strain tensor and stress tensor, respectively. The components of the two tensors are *S_i_* and *T_j_* (*i*, *j* = 1∼6). The components with subscripts *i*, *j* = 1∼3 indicate normal strains or normal stresses, while the components with subscripts *i*, *j* = 4∼6 indicate shear strains or shear stresses. ***D***, ***E*** are electric displacement vector and electric field strength, respectively. Their components are *D_k_* and *E_l_* (*k*, *l* = 1∼3). Subscripted numbers with the above symbols denote the related directions. By convention, 1, 2, 3 refer to the *x*, *y*, *z* directions, respectively, and 4, 5, 6 refer to the *y*-*z*, *x*-*z*, *x*-*y* directions. **s***^E^* is the elastic compliance tensor at constant electric field and its component *s_ij_* denotes the coefficient between *S_i_* and *T_j_*. ***ε****^T^* is the dielectric permittivity tensor at constant stress and its component *ε_kl_* denotes the coefficient between *D_k_* and *E_l_*. **d** is piezoelectric tensor constant and its component *d_kj_* denotes the coefficient between *D_k_* and *T_j_*. **d**^t^ is the transpose matrix of **d**.

Specifically, for quartz the **s***^E^*, **d** and ***ε**^T^* matrixes are found to be [19]

(3) $$s^{E}=\left(\begin{array}{cccccc} s_{11} & s_{12} & s_{13} & s_{14} & 0 & 0\\ s_{12} & s_{11} & s_{13} & -s_{14} & 0 & 0\\ s_{13} & s_{13} & s_{33} & 0 & 0 & 0\\ s_{14} & -s_{14} & 0 & s_{44} & 0 & 0\\ 0 & 0 & 0 & 0 & s_{44} & {2s}_{14}\\ 0 & 0 & 0 & 0 & {2s}_{14} & 2\left(s_{11}-s_{12}\right) \end{array}\right)$$

(4) $$d=\left(\begin{array}{cccccc} d_{11} & -d_{11} & 0 & d_{14} & 0 & 0\\ 0 & 0 & 0 & 0 & d_{14} & {-2d}_{11}\\ 0 & 0 & 0 & 0 & 0 & 0 \end{array}\right)$$

(5) $$\varepsilon^{T}=\left(\begin{array}{ccc} \varepsilon_{11} & 0 & 0\\ 0 & \varepsilon_{11} & 0\\ 0 & 0 & \varepsilon_{33} \end{array}\right)$$

**Figure 3.**
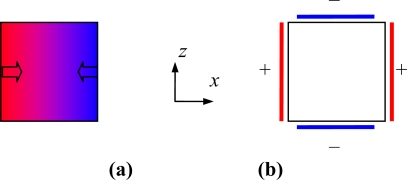
Schematic diagram of the drive beam’s cross section. **(a)** Strain distribution and electric field direction. **(b)** Electrode configuration corresponding to the electric field.

The drive vibration is designed to be produced by the bend of the drive beam, and is the action of normal compression strains and normal extension strains in the beam. Assuming that the drive beam bends in −*x* direction, the strain *S*_2_ distribution in the beam’s cross section is shown in Figure 3a [20]. The extension strain and compression strain are represented as the blue- and red-colored regions, respectively. According to (2), (3) and (4), the strain can be written as in (6).

(6) $$S_{2}=-d_{11}E_{1}+s_{12}T_{1}+s_{11}T_{2}+s_{13}T_{3}-s_{14}T_{4}$$

Therefore, the strain *S*_2_ can be excited by electric field *E*_1_. Corresponding to the strain distribution, the direction of *E*_1_ is shown in Figure 3a. Thus, a scheme of drive electrodes, shown in Figure 3b, is designed to produce the distribution of *E*_1_.

The sense beam vibrates along *y* direction and the dominant stresses in the beam are normal stress *T*_1_ and shear stress *T*_6_. According to (2), (4) and (5), by ignoring other stresses, ***D*** can be deduced as in (7).

(7) $$\left\{ \begin{array}{l} D_{1}=\varepsilon_{11}E_{1}+d_{11}T_{1}\\ D_{2}=\varepsilon_{11}E_{2}+d_{26}T_{6}\\ D_{3}=\varepsilon_{33}E_{3} \end{array}\right.$$

**Figure 4.**
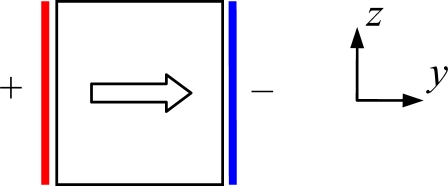
Electrodes configuration of sense beam.

Therefore, the sense beam’s vibration can generate electric displacement *D*_1_ and *D*_2_ under the action of *T*_1_ and *T*_6_. Compared with *D*_1_, *D*_2_ is easy to detect by setting a pair of electrodes on the +*y* and −*y* surfaces of the sense beam shown in Figure 4. Furthermore, the piezoelectric coefficient *d*_26_ = −2*d*_11_ on *T*_6_ is larger than *d*_11_ on *T*_1_ absolutely in (4). Thus, *T*_6_ can be detected more conveniently without excessive sensitivity loss.

According to the relation between electrodes and vibrations analyzed above, the overall electrodes, conducting wires, and pads are designed and have been shown in Figure 1. A sinusoidal voltage signal applied on drive pads can excite the drive vibration. And the sense vibration is transformed into charge signals that can be gathered by the sense electrodes.

### 2.3. Structure Design

Large inertial mass can improve gyroscopes’ performance efficiently [8,9,11,16]. In order to enlarge inertial mass, a pair of proof masses is attached to the ends of the drive beam. Reference [21] shows that the edge shear stress is zero for a beam of uniform cross section. Therefore, here the sense beam is designed to be a tapered beam to enhance the shear stress, *T*_6_ near the sense electrodes. The relationship between *T*_6_ and taper is deduced in detail as follows.

**Figure 5.**
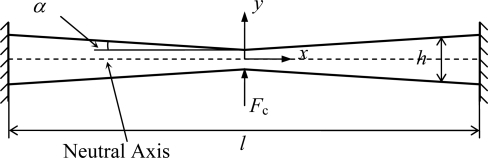
Schematic diagram of the sense beam.

The sense beam can be simplified as a symmetric tapered beam fixed at the two ends (shown in Figure 5). The beam’s height, length and width are *h*, *l* and *b,* respectively. Assume the beam’s taper of each side to be *α*, then

(8) $$h(x)=h_{0}+2\left|x\right|\cdot \text{tan}\;\alpha$$

where *h*_0_ is the height at the point *x* = 0. To simplify the deduction, only the right part of the beam (*x* > 0) is considered below. When force, *F*_c_, acts on the middle of the beam in *y* direction, the shear stress *T*_6_ in the beam is subjected to Equation (9) based on mechanics of materials [21].

(9) $$T_{6}(x,y)=\frac{V(x)Q(x)}{I(x)b}+\frac{M(x)h(x)}{2I(x)}\left(1-\frac{Q(x)h(x)}{I(x)}\right)\;\text{tan}\;\alpha$$

where *M*(*x*), *V*(*x*), *I*(*x*) and *Q*(*x*) are bending moment, shear force, inertia moment of the entire cross-sectional area computed about the neutral axis and first moment of the cross-sectional area above the level at which the shear stress *T*_6_ is being evaluated, at the point *x* respectively. They are expressed as in (10).

(10) $$\left\{ \begin{array}{l} V(x)=F_{c}/2\\ M(x)=\frac{F_{c}}{2}(l/4-x)\\ I(x)={\mathrm{bh}}^{3}(x)/12\\ Q(x)=\frac{b}{2}\left(\frac{h{\left(x\right)}^{2}}{4}-y^{2}\right) \end{array}\right.$$

According to (9) and (10), the edge shear stress (*y* = ±*h*/2) detected can be deduced to be

(11) $$T_{\text{edge}}=T_{6}(x,\pm h/2)=\frac{{3F}_{c}(l-4x)}{4b{\left(h_{0}+2x\;\text{tan}\;\alpha \right)}^{2}}\text{tan}\;\alpha$$

**Figure 6.**
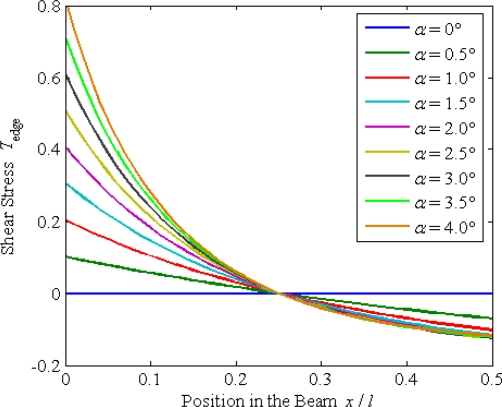
Edge shear stress distribution *versus* taper.

When *F*_c_ with a constant value is applied on the sense beam, the edge shear stress can be calculated using Equation (11). Different from the beam of uniform cross section, the edge shear stress is not zero for a tapered beam. Considering that the shear stress on the outside surface of a beam must be zero, a pair of electrodes needs to be covered on the sidewall surfaces of the beam. Therefore, the shear stress detected by the electrodes is the edge shear stress of the beam. According to Equation (11), the relationship between the edge shear stress *T*_edge_ distribution and the taper *α* is shown in Figure 6. The value of the edge shear stress *T*_edge_ is zero at the point of *x* = *l*/4. When *x* < *l*/4, the absolute value of *T*_edge_ increases with the taper *α*. However, it approaches a constant value in respect to *α* when *x* > *l*/4 and *α* > 1°. In other words, if the taper *α* is greater than 1°, the shear stress *T*_edge_ is insensitive to *α* at the position of *x* > *l*/4. Thus, in order to obtain uniform characteristics between different structures, the taper should be greater than 1 degree and the double ends quarter portions (*x* > *l*/4, *x* < −*l*/4) of the beam are used as sensing parts.

**Figure 7.**
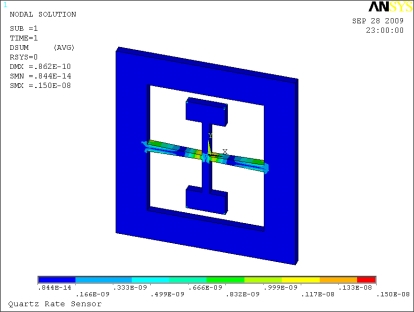
Electric displacement distribution by static analysis.

This shear stress detection scheme is simulated by ANSYS™ software. Static analysis is simulated with a force applied on the proof masses along the *y* direction. The result of electric displacement distribution produced by the sense beam’s deformation in *y* direction is shown in Figure 7. The electric displacement distribution corresponds to the shear stress distribution on the surface of the sense beam analyzed above, which validates the structure scheme designed.

The sensitivity of the sense beam can be defined as the ratio of the charges collected by the sense electrode to the Coriolis’ force. According to (7) and (11), the sensitivity can be deduced as

(12) $$S_{c}=\int_{A}\frac{{3d}_{26}(l-4x)\;\text{tan}\;\alpha }{4b{\left(h_{0}+2x\;\text{tan}\;\alpha \right)}^{2}}dxdz$$

where *A* is the area of the sense electrode. Thus *d*_26_ is a coefficient of *S_c_*.

**Figure 8.**
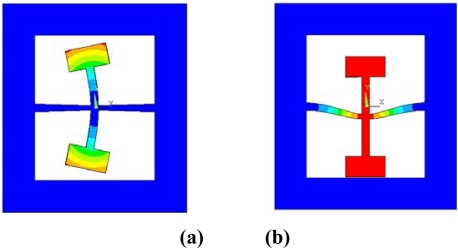
Mode analysis by ANSYS™. **(a)** Drive mode. **(b)** Sense mode.

Modal analyses are also simulated by changing the dimension of the entire structure to get the appropriate drive mode frequency and sense mode frequency. The modal analysis results are shown in Figure 8. The drive mode frequency is 13.286 kHz and the sense mode frequency is 13.538 kHz. The frequency difference between the drive mode and sense mode is about 250 Hz. This enables the structure to have sufficient sensitivity as well as enough robustness [22]. Table I summarizes the optimized structure parameters. In particular, the taper of the sense beam is designed as 1.64°.

The structure’s unique features gives this quartz gyroscope several advantages as follows:

- A pair of sense electrodes is formed on the sidewalls of the sense beam independently. This electrode layout is easy to be prepared by aperture masks.
- As both the drive mode and the sense mode are vibrating in the structure plane, air damping is slide-film damping. Therefore, it is easy to obtain a high quality factor.
- For quartz material, the piezoelectric coefficient *d*_26_ between shear stress *T*_6_ and electric displacement *D*_2_ is the largest of all, and the Coriolis’ force is detected by this coefficient. Thus, the sensor inherently has sufficient sensitivity.
- In order to increase the sensitivity of the gyroscope, a pair of proof masses is introduced and a tapered sense beam has been designed.

However, the structure is not a perfect design. For the drive beam’s asymmetric vibration, the main disadvantage is the poor sensitivity in response to accelerations. In order to overcome this problem, we will improve the structure design with a tuning fork instead of the cross-fork.

## 3. Fabrication

**Figure 9.**
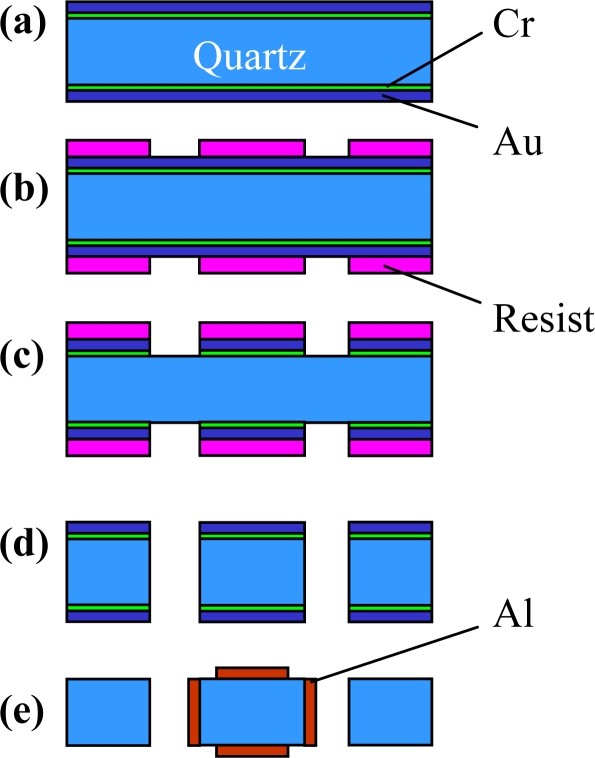
Cross-sectional scheme for the fabrication of the quartz gyroscope. **(a)** Films deposition. **(b)** Photolithography. **(c)** Etching mask films. **(d)** Etching quartz. **(e)** Preparation electrodes.

The prototype gyroscope was fabricated using a surface polishing z-cut quartz wafer with 500 μm thickness. The process combines anisotropic wet etching and vacuum evaporation technologies (shown in Figure 9). After the cleaning process, 100 Å Cr and 2,000 Å Au films are deposited on the surfaces of the wafer. A photolithography method is used to form the Cr/Au etching mask. Then, the quartz wafer is etched in a mixture of hydrofluoric acid (HF) and ammonium fluoride (NH_4_F) at 60 °C without stirring. The temperature greatly affects the etching rate. It is important to keep the temperature stable, otherwise the surface of the sidewalls of the beams will become rough. Normally, there are crystal facets on the surface of the -*x* plane after etching [23]. These crystal facets can be removed by increasing etching time.

**Figure 10.**
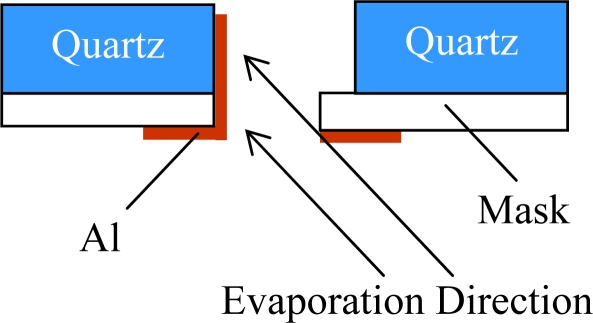
Fabrication of sidewall electrodes.

The electrode patterns and conducting wires are realized by direct evaporation of Al using an aperture mask method. Specifically, the fabrication of the sidewall electrodes needs special disposal. As the electrodes are on the sidewalls of the beams, the quartz wafer must be fixed with an angle to the evaporation direction (shown in Figure 10).

**Figure 11.**
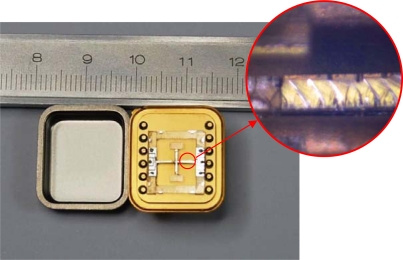
The photo of the prototype gyroscope.

The structure is mounted to a pedestal with epoxy, and electrically connected to the surrounding electronics with wire bonding. Figure 11 shows the fabricated device and the detail sidewall of the sense beam is shown in an enlarged view (see red circled area). The device’s dimension is 20.9 mm × 18.4 mm × 4.5 mm.

## 4. Readout Circuit

The readout circuit plays an important role in the overall performance of the gyroscope. It excites the drive beam to vibrate and convert extremely tiny charge signals into meaningful electrical signals. Moreover, the readout circuit demodulates the raw sense mode output, providing base-band signals proportional to the input angular rate.

**Figure 12.**
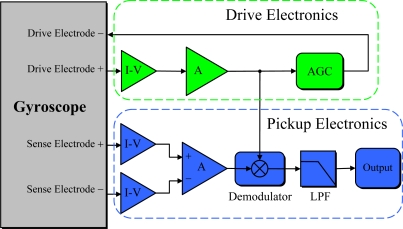
The block diagram of the readout circuit.

Figure 12 shows the block diagram of the proposed readout electronics. The drive electronics are required to maintain the resonance of the drive beam at constant amplitude. The drive beam, I–V converter, and amplifier consist of a resonator to excite the drive beam vibration at resonant frequency. In order to stabilize the drive beam’s vibration amplitude during gyroscope operation, an automatic gain controller (AGC) is introduced.

**Figure 13.**
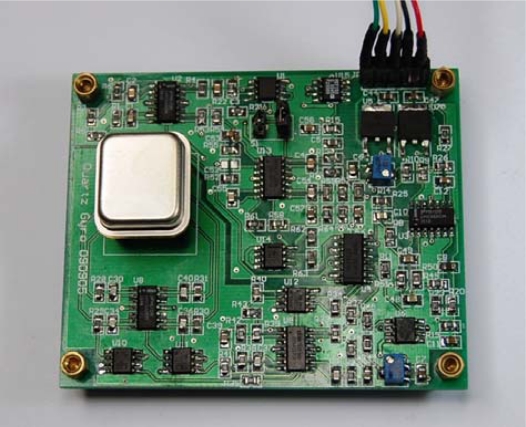
The photo of the readout circuit.

The pick-up electronics demodulate the rate signal from the sense charge signal. Changes of sense electrodes’ charges are differentially sensed by I–V converter. Then the signal is transferred to a high precision phase-sensitive demodulator, whose carrier signal is generated from the drive-mode electronics. High frequency signal components at the demodulator output are rejected by a high-order, low-pass filter, giving the base-band rate output. The photo of the readout circuit is shown in Figure 13.

## 5. Experimental Results

To present, experimental characterization of the gyroscope has included a mode test, Coriolis’ force test, and performance test.

### 5.1. Mode Test

**Figure 14.**
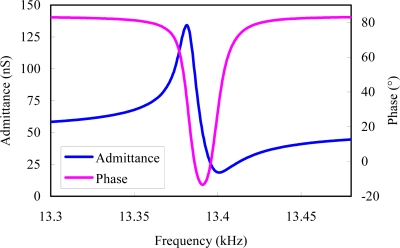
Admittance of drive mode.

**Figure 15.**
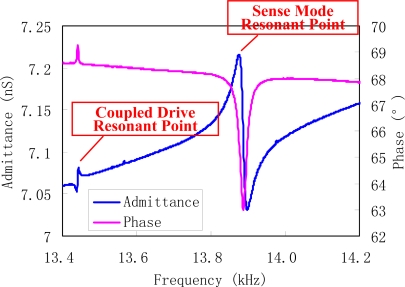
Admittance of sense mode.

A mode test is used to validate the vibration of the sensor’s modes. The drive resonator’s admittance is tested using a NF FRA5087 frequency response analyzer. From the admittance curves of drive mode (shown in Figure 14), the oscillator resonates at the frequency of 13.38 kHz approximately, and the quality factor deduced from the data is about 1,000. From this, we can conclude that the sensor can operate properly in an open-air environment without a vacuum package. The tested admittance of sense mode is shown in Figure 15. The two harmonic peaks, corresponding to the sense mode resonant point and the coupled drive resonant point can be observed. As voltages cannot effectively excite the shear strain of a beam by piezoelectric effect [24], the quality factor deduced from admittance data is very low. We are investigating an available method to characterize the sense beam excited by shear strain.

### 5.2. Coriolis’ Force Test

**Figure 16.**
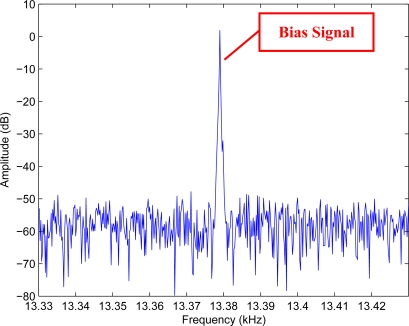
The frequency spectrum in case of no angular rate input.

**Figure 17.**
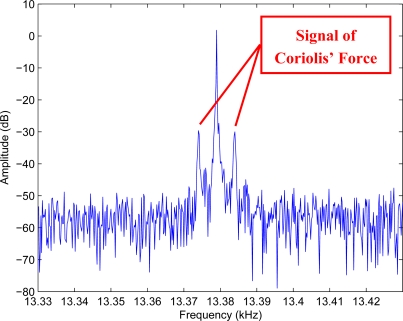
The frequency spectrum in case of angular rate input.

As micromachined gyroscopes measure angular rate signals by Coriolis effect, Coriolis’ force test can be used to validate the measurement ability of a gyroscope. Drive electrics excite the drive beam resonation and the output signal of the gyroscope is sampled by an Agilent infiniium DSO8104A oscilloscope. The Fast Fourier Transform (FFT) curves of the signal are shown in Figure 16. Thee spectral line at the drive frequency in the spectrum is bias signal. With a 4 Hz angular rate signal with amplitude of about 300 °/s input, there is a pair of clear spectral lines beside the central drive frequency shown in Figure 17, and this is the signal of the Coriolis’ force. This test validates the novel design scheme.

### 5.3. Performance Test

**Figure 18.**
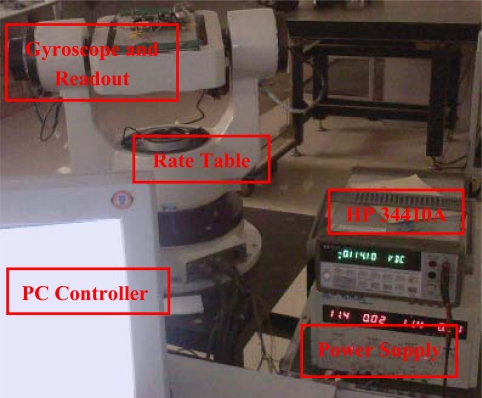
Experimental setup.

**Figure 19.**
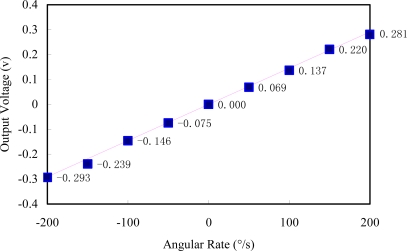
Output of the gyroscope *versus* the angular rate input.

As shown in Figure 18, a precision rate table is used to scale the gyroscope output. A PC controller controls the rotational speed of the rate table and a HP 34410A multimeter samples the output signal of the gyroscope. Figure 19 shows the output voltage under different input angular rates. The measured scale factor is 1.45 mV/°/s and the nonlinearity is 3.6% in full scale input range of ±200 °/s.

## 6. Conclusions

This paper presents the modeling, simulation, fabrication, and performance characterization of a novel z-axis quartz gyroscope. The proposed shear stress detection scheme simplifies the structure compared to traditional quartz gyroscopes. Specifically, we demonstrate that the sense electrodes do not need to be divided into two parts on each sidewall anymore. Therefore, the sensor’s electrodes are easy to fabricate and the structure can be miniaturized easily. In order to increase the sensor’s sensitivity, a pair of proof masses is attached to the ends of the drive beam, and the sense beam has a tapered design. The structure is etched from a z-cut quartz wafer. The drive mode frequency is 13.38 kHz, and the quality factor is about 1,000 in air atmosphere pressure. Therefore, the gyroscope can work in air without a vacuum package. The experimentally obtained scale factor is 1.45 mV/°/s and the nonlinearity is 3.6% in range of ±200 °/s.

## Figures and Tables

**Table 1.** Structure parameters of the device.

Parameter	Value
Thickness (mm)	0.50
Mass width (mm)	1.30
Mass length (mm)	2.50
Drive beam width (mm)	0.48
Drive beam length (mm)	5.00
Sense beam length (mm)	7.48
Sense beam width (mm)	0.50
Sense beam taper (degree)	1.64

## References

[1] Dixon R.H., Bouchaud J. (2006). Markets and applications for MEMS inertial sensors. Proc. SPIE.

[2] Weinberg M.S., Kourepenis A. (2006). Error sources in in-plane silicon tuning-fork MEMS gyroscopes. J. Microelectromech. Syst.

[3] Choi B., Lee S.Y., Kim T., Baek S.S. (2009). Dynamic characteristics of vertically coupled structures and the design of a decoupled micro gyroscope. Sensors.

[4] Closkey R.M., Challoner A.D. Modeling, identification, and control of micro-sensor prototypes.

[5] Soderkvist J. (1996). Micromachined vibrating gyroscopes. Proc. SPIE.

[6] Megherbi S., Levy R., Parrain F., Mathias H., Traon O.L., Janiaud D., Gilles J.P. Behavioral modelling of vibrating piezoelectric micro-gyro sensor and detection electronics.

[7] Jaffe R., Simshauser S., Madni A.M. Quartz dual axis rate sensor.

[8] Kikuchi T., Gouji S., Tai T., Hayashi S., Okada N., Tani M., Ishikawa S., Yokoi S., Enokijima T., Kawamura Y. Miniaturized quartz vibratory gyrosensor with hammer-headed arms.

[9] Madni A.M., Costlow L.E., Smith M.W. The μGyro: a quartz MEMS automotive gyroscope.

[10] Gupta P.K., Jenson C.E. Rotation rate sensor with center mounted tuning fork.

[11] Knowles S.J. Inertial rate sensor tuning fork.

[12] Soderkvist J. (1991). Piezoelectric beams and vibrating angular rate sensors. IEEE Trans. Ultrason. Ferroelectr. Freq. Contr.

[13] Madni A.M., Wan L.A., Hammons S. A microelectromechanical quartz rotational rate sensor for inertial applications.

[14] Senturia S.D. (2000). Microsystem Design.

[15] Uehara H., Ohtsuka T., Inoue T. Miniaturized angular rate sensor with laminated quartz tuning fork.

[16] Ohtsuka T., Inoue T., Yoshimatsu M., Matsudo H., Okazaki M. Development of an ultra-small angular rate sensor element with a laminated quartz tuning fork.

[17] Sato K., Ono A., Tomikawa Y. (2004). Experimental study of gyro sensor using double-ended tuning fork quartz resonator. Jpn. J. Appl. Phys.

[18] Soderkvist J. (1990). An analysis of space-dependent electric fields used in exciting flexural vibartions of piezoelectric beams. Meas. Sci. Technol.

[19] Qin Z.K. (1980). Piezoelectric Quartz Crystal.

[20] Hibbeler R.C., George D.A. (2005). Mechanics of Materials.

[21] Timoshenko S.P., Gere J.M. (1973). Mechanics of Materials.

[22] Schofield A.R., Trusov A.A., Shkel A.M. Design trade-offs of micromachined gyroscope concept allowing interchangeable operation in both robust and precision modes.

[23] Rangsten P., Hedlund C., Katardjiev I.V., Backlund Y. (1998). Etch rates of crystallographic planes in z-cut quartz—experiments and simulation. J. Micromech. Microeng.

[24] Wang J.F. (1989). Piezoelectric Vibration.

